# Positive health during the COVID-19 pandemic: a survey among community-dwelling older individuals in the Netherlands

**DOI:** 10.1186/s12877-021-02737-2

**Published:** 2022-01-13

**Authors:** I. S. Moens, L. J. van Gerven, S. M. Debeij, C. H. Bakker, M. J. C. Moester, S. P. Mooijaart, S. van der Pas, M. Vangeel, J. Gussekloo, Y. M. Drewes, W. P. J.den Elzen

**Affiliations:** 1 Department of Internal Medicine, Section of Gerontology and Geriatrics, LUMC, Leiden, Netherlands; 2 Master Vitality and Ageing, Department of Internal Medicine Section, Gerontology and Geriatrics, LUMC, Leiden, Netherlands; 3 Biomedical Data Sciences, LUMC, Leiden, Netherlands; 4 Department of Public Health and Primary Care, Faculty of Social Work & Applied Psychology, University of Applied Sciences Leiden, Leiden, Netherlands; 5 Master Vitality and Ageing, Department of Internal Medicine, Section of Gerontology and Geriatrics, Department of Public Health and Primary Care, LUMC, Leiden, Netherlands; 6 Department of Internal Medicine Section Gerontology and Geriatrics, LUMC, Mailbox 9600, Postal zone C7-Q, route 221, 2300 RC Leiden, Netherlands; 7 Clinical Atalmedial Diagnostics Centre, Amsterdam & Amsterdam UMC, Amsterdam, Netherlands; 8 Department of Clinical Chemistry, Amsterdam Public Health Research Institute, Amsterdam, The Netherlands

**Keywords:** Online questionnaire, Bodily functions, Mental well-being, Meaningfulness, Quality of life, Social participation, Daily functioning, COVID-19

## Abstract

**Background:**

Coronavirus Disease 2019 (COVID-19) reached the Netherlands in February 2020. To minimize the spread of the virus, the Dutch government announced an “intelligent lockdown”. Older individuals were urged to socially isolate completely, because they are at risk of a severe disease course. Although isolation reduces the medical impact of the virus, the non-medical impact should also be considered.

**Aim:**

To investigate the impact of COVID-19 pandemic and associated restrictive measures on the six dimensions of Positive Health in community-dwelling older individuals living in the Netherlands, and to identify differences within subgroups.

**Methods:**

In May/June 2020, community-dwelling older individuals aged ≥ 65 years completed an online survey based on Huber’s model of Positive Health. Positive Health was measured regarding the appreciation of the six dimensions (categorized as poor/satisfactory/excellent) and a comparison with a year before (categorized as decreased/unchanged/increased) using frequencies (%) and a chi-square test.

**Results:**

834 older individuals participated (51% women, 38% aged ≥ 76 years, 35% living alone, 16% self-rated poor health). Most respondents assessed their bodily functions, mental well-being and daily functioning as satisfactory, their meaningfulness and quality of life (QoL) as excellent, and their social participation as poor. 12% of the respondents reported a deterioration of 4–6 dimensions and 73% in 1–3 dimensions, compared to the past year. Deterioration was most frequently experienced in the dimension social participation (73%), the dimension mental well-being was most frequently improved (37%) and quality of life was in 71% rated as unchanged. Women more often observed a deterioration of 4–6 dimensions than men (15% vs. 8%, *p* = 0.001), and individuals with self-rated poor health more often than individuals with self-rated good health (22% vs. 10%, *p* < 0.001). Older individuals living alone experienced more frequently a decrease in meaningfulness compared to older individuals living together.

**Conclusion:**

The COVID-19 pandemic and associated restrictive measures had a substantial impact on all six dimensions of Positive Health in community-dwelling older individuals, especially in women, respondents living alone and respondents with self-rated poor general health.

**Supplementary Information:**

The online version contains supplementary material available at 10.1186/s12877-021-02737-2.

## Background

In February 2020 the first case of COVID-19, caused by the Severe Acute Respiratory Syndrome Coronavirus-2 (SARS-CoV-2), was identified in the Netherlands [1]. In March, the outbreak of this Coronavirus was declared a pandemic by the WHO [2]. To reduce the spread of the SARS-CoV-2, the Dutch government urged its citizens to adhere to the rules of the so-called “intelligent lockdown”, in which the population of the Netherlands is advised to keep 1.5 m distance from each other, minimize contact and self-isolate at home [3]. In the Netherlands, 90.5% of all COVID-19 mortality occurred in people aged 70 years or older [4]. Therefore, the government urged especially older people to fully comply to the lockdown rules [5, 6].

Since the discovery of SARS-CoV-2, multiple studies have been conducted to clarify the medical impact (adverse symptoms, hospitalization, mortality rate) of this virus on older individuals [7–9]. It recently has been shown that especially older individuals with chronic conditions have higher odds of being hospitalized due to COVID-19 [10]. With this knowledge, the importance for them to maintain healthy lifestyles and a good general health is underlined. Unfortunately, the COVID-19 pandemic could also have a non-medical impact on the lives of older individuals. Getting infected with COVID-19, experiencing the symptoms and the restrictions and the possibility of hospitalization could provoke adverse mental health outcomes such as anxiety, stress and depression [11, 12]. Both the medical and non-medical dimensions are captured in the new definition of health which M. Huber proposed in 2011: ‘’Health as the ability to adapt and to self-manage, in the face of social, physical and emotional challenges” [13]. The Positive Health Model is developed to make this definition measurable and covers six medical and non-medical dimensions: bodily functions, mental well-being, meaningfulness, quality of life, social participation and daily functioning [14].

Furthermore, studies on the impact of the COVID-19 pandemic are mainly focusing on institutionalized older individuals or those who are admitted to the hospital, ignoring the three million community-dwelling older persons aged 65 and over living in the Netherlands [15–19].

## Methods

The present study aims to gain insight into the impact of the COVID-19 pandemic and associated preventive restrictions on the six dimensions of Positive Health in community-dwelling older individuals (65 +) living in the Netherlands, and to identify differences within the subgroups sex, age, living situation and self-rated general health.

### Respondents

This study is embedded in the “Positive Health Impact of the COVID-19 pandemic and the associated measures on community-dwelling Older individuals and Professionals study” (PHICOP). With an online survey we investigated cross-sectionally the impact of the COVID-19 pandemic and associated restrictive measures on all six dimensions of Huber’s model of Positive Health in community-dwelling older persons living in the Netherlands.

Between 11 May and 15 June 2020, thus 2 months after the implementation of the measures, community-dwelling older persons aged ≥ 65 years living in the Netherlands were invited to participate in the online survey. Older individuals living in nursing homes or other institutionalized care forms were excluded from participation. Recruitment took place through e-mails via welfare organizations, senior organizations and social media, which led to the desired snowball effect. Questionnaires with < 98% completed questions were excluded in order to obtain full information about all dimensions and to prevent inclusion of repeated entries by the same individuals, as some individuals experienced technical issues after opening the questionnaire. Furthermore, duplicated questionnaires were excluded.

### Measures

Positive Health was measured by two questions on the appreciation of bodily functions, mental well-being, meaningfulness, quality of life, social participation and daily functioning. The first question being: “How do you assess your [dimension] currently?”. The second question was: “Compared to the past year, how would you assess your [dimension] currently?”. For the analysis of current appreciation of the dimensions, the answer categories were merged into three categories namely: “poor”, “satisfactory”, “excellent”. For the analysis of self-rated change, the answer categories were merged into three categories namely: “decreased”, “unchanged”, “increased”. Detailed information on the original response categories and conversion of the response categories for data analysis is provided in the Additional file 1. The questions of this questionnaire were based on or retrieved from the validated questionnaire of the Institute for Positive Health [14, 20]. Furthermore, additional information regarding sex, age, educational level, living situation, care use, contact with family and general health was obtained.

### Data analysis

The categorical data were presented as proportions with their corresponding 95% intervals. P-values were obtained by chi-square tests. In all tests a p-value < 0.05 was considered statistically significant. The dimensions that respondents marked as “decreased” were summed to calculate the total number of negatively affected dimensions. With subgroup analyses, results were stratified for sex, age (65–75/ ≥ 76 years), living situation (alone/with others), and self-rated general health (poor/good). The data obtained by the questionnaires were analyzed using IBM SPSS statistics 25.

## Results

### Participants’ characteristics

Of the 837 surveys with ≥ 98% of the questions answered, two were excluded because they were duplicates, and one was excluded because the participant lived in a nursing home. Finally, 834 completed questionnaires were included for analysis. Of these 834 participants, 51% was female, 38% was aged ≥ 76 years, 35% lived alone and 16% suffered self-rated poor general health (Table 1).

**Table 1:** Characteristics of the 834 community-dwelling older participants living in the Netherlands

		**n (%)**
Women ^a^		427 (51)
Aged 76 years and over		317 (38)
Lower educated ^b^		318 (38)
Care use ^c^		202 (24)
Living alone		290 (35)
Urban living		733 (88)
Contact with children		695 (83)
Contact with siblings		658 (79)
Self-rated poor general health		134 (16)
*Self-perceived appreciation of the six dimensions*		
Bodily functions	Grade < 6.0	46 (5.5)
	Grade 6.0–8.0	591 (71)
	Grade > 8.0	197 (24)
Mental wellbeing	Grade < 6.0	60 (7.2)
	Grade 6.0–8.0	630 (76)
	Grade > 8.0	144 (17)
Meaningfulness ^d^	Poor	68 (8.1)
	Satisfactory	227 (27)
	Excellent	530 (64)
Quality of life ^e^	Poor	43 (5.1)
	Satisfactory	351 (42)
	Excellent	436 (52)
Social participation ^f^	Poor	417 (50)
	Satisfactory	288 (35)
	Excellent	126 (15)
Daily functioning ^g^	Poor	61 (7.3)
	Satisfactory	487 (58)
	Excellent	284 (34)

^a^22 missing (2.6%)

^b^Lower educated: participants who did not receive “higher professional education” or “university education”

^c^Care use: participants who are being treated by/receiving help from a physiotherapist, psychologist or homecare

^d^9 no opinion (1.1%)

^e^4 no opinion (0.5%)

^f^3 no opinion (0.4%)

^g^2 no opinion (0.2%)

### Positive Health: appreciation at the moment of filling out the questionnaire

When summing the number of dimensions affected per respondent, 12.0% of the respondents experienced a deterioration of 4 to 6 dimensions during the COVID-19 pandemic. Furthermore, 72.7% of the respondents reported 1 to 3 negatively affected dimensions and 15.4% of the older individuals did not experience a decrease in any of the dimensions.

For the self-rated dimension appreciation at the moment of filling out the questionnaire, most respondents scored “satisfactory” on the dimensions bodily functions (70.8%), mental wellbeing (75.6%) and daily functioning (58.4%). “Excellent” was most selected for the dimensions meaningfulness (63.5%) and quality of life (52.3%). Lastly, 50.0% of the respondents rated their social participation as “poor” (Table 1).

### Positive Health: self-rated change in dimensions compared to one year ago

**Fig. 1:**
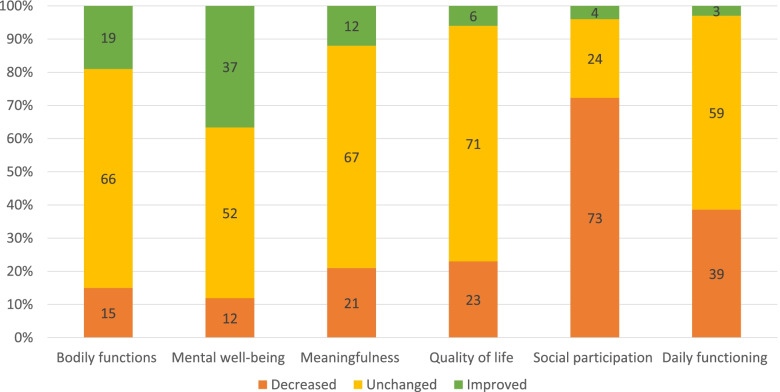
Overall self-rated change in the six Positive Health dimensions in Dutch older individuals (*n* = 834)

All dimensions considered, 23.5% to 70.9% of the respondents did not observe a change in the corresponding dimension (Fig. 1 and Additional file 2).

In five of the six dimensions, namely bodily functions, mental well-being, meaningfulness, quality of life and daily functioning, more than half of the respondents rated the dimension as unchanged. The least change was observed in the dimension quality of life where 591 respondents (70.9%) rated it as unchanged. The dimension social participation was most negatively affected with 72.5% of the respondents noticing a decline, whereas mental well-being was the most positively impacted dimension with 36.8% of the individuals noticing an improvement.

### Stratified analyses

#### Sex

Women more often than men reported a deterioration of 4 to 6 dimensions (15.3% vs. 8.4%, *p* = 0.001). Additionally, men more often reported that none of the dimensions had deteriorated (18.7% vs. 12.2%, *p* = 0.001) (Additional file 3).

Women more often than men reported a decrease in the dimensions bodily functions (Table 2 and Additional file 3, 18.7% vs. 10.4%, *p* = 0.004), mental wellbeing (14.3% vs. 9.1%, *p* = 0.01), meaningfulness (26.5% vs. 14.9%, *p* < 0.001), quality of life (26.1% vs. 20.1%, *p* = 0.001) and social participation (76.3% vs 69.0%, *p* = 0.006). Furthermore, more women seemed to experience an improvement in mental well-being (38.6% vs. 34.3%, *p* = 0.01), meaningfulness (13.0% vs. 10.2%, *p* < 0.001), quality of life (8.0% vs. 3.1%, *p* = 0.001) and social participation (4.7% vs. 2.9%, *p* = 0.006). No difference between sexes was observed in daily functioning.

**Table 2:** Self-rated change in the six dimensions of Positive Health compared to before the COVID-19 pandemic

			**Decreased % (n)**	**Unchanged % (n)**	**Improved % (n)**	**p-value**
Sex	Bodily functions	Men	10.4 (40)	69.9 (269)	19.7 (76)	0.004
		Women	18.7 (80)	63.2 (270)	18.0 (77)	
	Mental well-being	Men	9.1 (35)	56.6 (218)	34.3 (132)	0.010
		Women	14.3 (61)	47.1 (201)	38.6 (165)	
	Meaningfulness	Men	14.9 (57)	74.9 (287)	10.2 (39)	< 0.001
		Women	26.5 (112)	60.4 (255)	13.0 (55)	
	Quality of life	Men	20.1 (77)	76.8 (295)	3.1 (12)	0.001
		Women	26.1 (111)	66.0 (281)	8.0 (34)	
	Social participation	Men	69.0 (263)	28.1 (107)	2.9 (11)	0.006
		Women	76.3 (325)	19.0 (81)	4.7 (20)	
	Daily functioning	Men	37.6 (144)	61.1 (234)	1.3 (5)	0.088
		Women	39.7 (169)	56.8 (242)	3.5 (15)	
Age	Bodily functions	65–75 years	15.7 (81)	67.9 (351)	16.4 (85)	0.061
		76 years and over	13.9 (44)	63.1 (200)	23.0 (73)	
	Mental well-being	65–75 years	12.0 (62)	50.3 (260)	37.7 (195)	0.644
		76 years and over	11.0 (35)	53.6 (170)	35.3 (112)	
	Meaningfulness	65–75 years	22.8 (117)	64.9 (333)	12.3 (63)	0.192
		76 years and over	18.8 (59)	71.0 (223)	10.2 (32)	
	Quality of life	65–75 years	23.1 (119)	70.5 (363)	6.4 (33)	0.602
		76 years and over	23.7 (75)	71.6 (227)	4.7 (15)	
	Social participation	65–75 years	74.5 (383)	21.4 (110)	4.1 (21)	0.184
		76 years and over	69.2 (218)	27.0 (85)	3.8 (12)	
	Daily functioning	65–75 years	38.7 (199)	58.4 (300)	2.9 (15)	0.647
		76 years and over	38.5 (122)	59.6 (189)	1.9 (6)	
Living situation	Bodily functions	Alone	17.9 (52)	61.0 (177)	21.0 (61)	0.050
		With others	13.0 (70)	69.1 (372)	17.8 (96)	
	Mental well-being	Alone	13.8 (40)	52.4 (152)	33.8 (98)	0.228
		With others	10.6 (57)	50.7 (273)	38.7 (208)	
	Meaningfulness	Alone	27.6 (79)	58.7 (168)	13.6 (39)	0.001
		With others	17.9 (96)	71.8 (384)	10.3 (55)	
	Quality of life	Alone	26.6 (77)	66.6 (193)	6.9 (20)	0.151
		With others	21.8 (117)	72.9 (391)	5.2 (28)	
	Social participation	Alone	69.2 (200)	25.6 (74)	5.2 (15)	0.227
		With others	74.2 (396)	22.5 (120)	3.4 (18)	
	Daily functioning	Alone	40.0 (116)	56.2 (163)	3.8 (11)	0.178
		With others	37.9 (203)	60.2 (322)	1.9 (10)	
Self-rated general health	Bodily functions	Poor	21.6 (29)	43.3 (58)	35.1 (47)	< 0.001
		Good	13.7 (96)	70.4 (493)	15.9 (111)	
	Mental well-being	Poor	17.9 (24)	38.8 (52)	43.3 (58)	0.002
		Good	10.4 (73)	54.0 (378)	35.6 (249)	
	Meaningfulness	Poor	28.8 (38)	57.6 (76)	13.6 (18)	0.031
		Good	19.9 (138)	69.1 (480)	11.1 (77)	
	Quality of life	Poor	39.6 (53)	56.7 (76)	3.7 (5)	< 0.001
		Good	20.2 (141)	73.6 (514)	6.2 (43)	
	Social participation	Poor	75.2 (100)	20.3 (27)	4.5 (6)	0.616
		Good	72.0 (501)	24.1 (168)	3.9 (27)	
	Daily functioning	Poor	54.9 (73)	39.1 (52)	6.0 (8)	< 0.001
		Good	35.5 (248)	62.6 (437)	1.9 (13)	

#### Age

No differences were observed in the number of deteriorated dimensions and change in the six dimensions between participants aged 65 to 75 years and participants aged 76 years and over.

#### Living situation

Between participants living alone and participants living with others, no differences were observed in the number of deteriorated dimensions.

Living situation seemed to be of importance only for the dimension meaningfulness. A decline in this dimension was more frequently present in individuals living alone compared to individuals living together (27.6% vs. 17.9%, *p* = 0.001). Likewise, an improvement was more common in individuals living alone (13.6% vs. 10.3%, *p* = 0.001).

#### Self-rated general health

Substantially more participants with self-rated poor health noticed a deterioration in 4 to 6 of the dimensions compared to participants with self-rated good health (21.5% vs. 10.1%, *p* < 0.001). Additionally, fewer participants with self-rated poor health observed no deterioration in any of the dimensions compared to those with self-rated good health (9.2% vs. 16.5%, *p* < 0.001).

Individuals with self-rated poor health more frequently reported a decrease in their bodily functions (21.6% vs. 13.7%, *p* < 0.001), mental well-being (17.9% vs. 10.4%, *p* = 0.002), meaningfulness (28.8% vs. 19.9%, *p* = 0.031), quality of life (39.6% vs. 20.2%, *p* < 0.001) and daily functioning (54.9% vs. 35.5%, *p* < 0.001) than respondents with self-rated good health. Also, the respondents with self-rated poor health more often reported an improvement of the dimensions bodily functions (35.1% vs. 15.9%, *p* < 0.001), mental well-being (43.3% vs. 35.6%, *p* = 0.002), meaningfulness (13.6% vs. 11.1%, *p* = 0.031) and daily functioning (6.0% vs. 1.9%, *p* < 0.001) than the individuals with self-rated good health.

## Discussion

### Main findings

This study investigated the impact of the COVID-19 pandemic and associated preventive measures on the six dimensions of Positive Health in community-dwelling older individuals living in the Netherlands. The dimensions bodily functions, mental well-being and daily functioning were most rated as being satisfactory at the moment of filling out the questionnaire. Meaningfulness and quality of life were mostly rated as excellent, and social participation mostly as poor. In 12.0% of the older individuals 4 to 6 dimensions were negatively affected and in 72.7% of the respondents 1 to 3 dimensions. Participants reported a substantial decline in social participation. All other five dimensions were (negatively) affected as well, but the majority of the respondents did not notice a change in those dimensions. Particularly women, respondents who lived alone and older individuals with self-rated poor health observed a decline in dimensions. Women seemed to be more at risk for experiencing a decrease in their level of bodily functions, mental well-being, meaningfulness, quality of life and social participation during the COVID-19 pandemic. Furthermore, people who lived alone were prone to experiencing a decline in meaningfulness. Lastly, more people with self-rated poor health showed a decrease in bodily functions, mental well-being, meaningfulness, quality of life and daily functioning. Overall, mental well-being was the most positively impacted dimension with 36.8% of the respondents noticing an improvement.

### Comparison with existing research

During the COVID-19 pandemic, every country implemented a combination of different restrictions. Therefore, the experienced effects of the COVID-19 pandemic could differ per population as well. The participants of our study experienced the various restrictions of the Dutch “intelligent lockdown”. Previous studies dedicated to the consequences of COVID-restrictions on community-dwelling older individuals show that all these restrictions of the Dutch intelligent lockdown could have their impact on the lives of the older individuals. A study from Wang et al. showed that citizens of countries that are not used to wearing face masks are more prone to having physical and mental complaints compared to citizens of countries that are familiar with it [21]. However our findings show that, although the Dutch older individuals were not used to wearing face masks, the majority did not notice a change in their physical and mental health. Our findings are in line with the results of a study which found that the level of depression, stress, and anxiety observed has been low during a partial lockdown [22]. However, in line with recently published studies, our study did show that social isolation places some of the older individuals at risk for disadvantageous outcomes on mental well-being, such as depression-like symptoms and anxiety [23, 24]. In accordance with the situation prior to the COVID-19 pandemic, where women showed to be more prone to depression than men, our study showed women to be more at risk than men of experiencing a decreased mental-wellbeing, meaningfulness and quality of life than before the COVID-19 pandemic [25]. This finding corresponds with a recently published article about the psychological effects of the COVID-19 pandemic on women in Iran [26]. In that study, women were described as a vulnerable group for the stressors of the pandemic because they are more prone to mental disorders such as anxiety and depression and because of their multifaceted responsibilities including being a (grand)mother. Further research is needed into the vulnerable position of women during a pandemic to develop possible interventions. In contrast with other studies that showed only negative effects of the COVID-19 pandemic and SARS-outbreak on mental well-being and psychological reliance in older persons, we found the mental well-being improved in more than one third of the older individuals [27–29]. This finding is in line with the research conducted by telephone by Brown et al. in which the older individuals reported low levels of mental problems during COVID-19 [30]. Furthermore, the Dutch government implemented the “intelligent lockdown” promptly which could clarify that the prevalence of depressive symptoms was low [31].

Our findings also suggest that individuals who lived alone and individuals with self-rated poor health have a high risk of experiencing less meaning in life during the COVID-19 pandemic, indicating that meaningfulness should be monitored during a pandemic with restrictive measures. These results build on evidence from studies which have previously established the importance of good health, close relationships and connectedness for the well-being and purpose of life in older individuals [32–34].

In addition, the results of this study show that more women than men experienced a decreased social participation, despite the fact that both sexes participated approximately equally in 2019 [35]. The explanation for this is yet unclear, but may involve differences in types of activities [35, 36] or level of fear of getting infected with the coronavirus.

### Strengths and limitations

The current research has several strengths. First, the online surveys allowed us to easily reach a large sample of older individuals across the country. Secondly, the respondents were recruited during the first months of the pandemic. This allowed us to study the change of impact during the pandemic. Third, the anonymity and online accessibility of the survey may have lowered the threshold to participate. Lastly, the questions of the questionnaire were based on a validated questionnaire on the Positive Health Model which has already been implemented in the Netherlands [37, 38].

A number of limitations also have to be acknowledged. First, the generalizability of our study sample is important to address. Because we used an online survey, only the individuals with internet skills and (financial) access to the internet were able to participate. Second, the cross-sectional design of the study and the fact we asked participants during the pandemic on their view of the situation before the pandemic hamper definite conclusions about causality. Third, since a snowball method was used, selection bias could have occurred. However, according to the baseline characteristics a variety of older people is presented in this survey. Fourth, since the data were only stratified for basic variables, there is a risk for unmeasured confounding. Therefore, we mainly focused on a descriptive analysis in the result section. Last, although the data were gathered two months after the first wave of the COVID-19 pandemic, consecutive events occurred in a short time period and recall bias could have occurred. However, our findings offer important early insights for further research, which should include a more heterogenous group of older individuals with different socio-economic backgrounds, health status and internet skills. In addition, our research should be repeated to investigate the long-term effects of the COVID-19 pandemic.

To conclude, this research aimed to examine the impact of the COVID-19 pandemic and associated preventive measures on the six dimensions of Positive Health in community-dwelling older individuals living in the Netherlands. In numerous older individuals one or more of these dimensions were affected negatively, especially in women, respondents who lived alone and older individuals with self-rated poor health. Our results suggest that, in future waves of the COVID-19 pandemic and in future comparable crises, a balance should be achieved between medical protection with social restriction, and the impact on medical and non-medical health in older individuals. With this research being one of the first on this topic, our results offer a foundation for policymakers and further research.

## Supplementary Information

**Additional file 1.** Original response categories and conversion of the response categories for data analysis.

**Additional file 2.** Self-rated change in the six dimensions of Positive Health compared to the year before the COVID-19 pandemic in older individuals living in the Netherlands (*n*=834).

**Additional file 3.** Self-rated change (%), compared to before the COVID-19 pandemic, in the six dimensions of Positive Health of older individuals living in the Netherlands depending on sex, age, living situation and self-rated general health (*n*=834). * = statistically significant.

## Data Availability

The datasets used and/or analysed during the current study are available from the corresponding author on reasonable request.

## Abbreviations

COVID-19: Coronavirus Disease 2019
QoL: Quality of Life
SARS-CoV-2: Severe Acute Respiratory Syndrome Coronavirus-2
