# Supplementary material for: Positive health during the COVID-19 pandemic: a survey among community-dwelling older individuals in the Netherlands
Source: BMC Geriatr. 2022 Jan 13;22:51. doi: 10.1186/s12877-021-02737-2 (PMC8756757; doi:10.1186/s12877-021-02737-2)
Supplement: Supplementary file 1 — Additional file 1. Original response categories and conversion of the response categories for data analysis. [file 12877_2021_2737_MOESM1_ESM.docx]

**Additional file 1**

| Original response categories and conversion of the response categories for data analysis | | | |
| --- | --- | --- | --- |
| *Question* | ***Dimension*** | ***Original response categories*** | ***Converted response categories*** |
| How do you asses your [dimension] currently? | Bodily functions  Mental well-being | Grade (0-10, 0.5 intervals) | Poor = <6.0  Satisfactory = 6.0-8.0  Excellent = >8.0 |
|  | Meaningfulness  Quality of life  Social participation  Daily functioning | Very poor, poor, satisfactory, good, excellent and no opinion | Poor = very poor & poor  Satisfactory = satisfactory  Excellent = very good & excellent  *The answer category “no opinion” was not included for analysis.* |
| Compared to the past year, how would you assess your [dimension] currently | Bodily functions  Mental well-being | Grade (0-10, 0.5 intervals) | The difference in grade before the pandemic and during the pandemic was calculated to investigate if the domain had decreased, remained unchanged, or increased. |
|  | Meaningfulness  Quality of life  Social participation  Daily functioning | Much worse, slightly worse, unchanged, slightly better, much better, and no opinion | Decreased = much worse & worse  Unchanged = unchanged  Increased = slightly better & much better  *The answer category “no opinion” was not included for analysis.* |
| Overall, how do you asses your health? |  | Poor, moderate, good, very good, excellent, and no opinion. | Poor = poor & moderate  Good = good, very good & excellent  *The answer category “no opinion” was not included for analysis.* |
