# Supplementary material for: Positive health during the COVID-19 pandemic: a survey among community-dwelling older individuals in the Netherlands
Source: BMC Geriatr. 2022 Jan 13;22:51. doi: 10.1186/s12877-021-02737-2 (PMC8756757; doi:10.1186/s12877-021-02737-2)
Supplement: Supplementary file 2 — Additional file 2. Self-rated change in the six dimensions of Positive Health compared to the year before the COVID-19 pandemic in older individuals living in the Netherlands (n=834). [file 12877_2021_2737_MOESM2_ESM.docx]

**Additional file 2**

| Self-rated change in the six dimensions of Positive Health compared to the year before the COVID-19 pandemic in older individuals living in the Netherlands (n=834) | | | | | | |
| --- | --- | --- | --- | --- | --- | --- |
|  | **Decreased** | | **Unchanged** | | **Improved** | |
|  | **n** | **% (95% CI)** | **n** | **% (95% CI)** | **n** | **% (95% CI)** |
| Bodily functions | 125 | 15 (13-17) | 551 | 66 (63-69) | 158 | 19 (16-22) |
| Mental well-being | 97 | 12 (9-14) | 430 | 52 (48-55) | 307 | 37 (34-40) |
| Meaningfulness | 176 | 21 (19-24) | 556 | 67 (64-70) | 95 | 12 (9-14) |
| Quality of life | 194 | 23 (20-26) | 590 | 71 (68-74) | 48 | 6 (4-7) |
| Social participation | 601 | 73 (70-76) | 195 | 24 (21-26) | 33 | 4 (3-5) |
| Daily functioning | 321 | 39 (35-42) | 489 | 59 (55-62) | 21 | 3 (2-4) |
