# Supplementary material for: Positive health during the COVID-19 pandemic: a survey among community-dwelling older individuals in the Netherlands
Source: BMC Geriatr. 2022 Jan 13;22:51. doi: 10.1186/s12877-021-02737-2 (PMC8756757; doi:10.1186/s12877-021-02737-2)

**Additional file 3:** Self-rated change (%), compared to before the COVID-19 pandemic, in the six dimensions of Positive Health of older individuals living in the Netherlands depending on sex, age, living situation and self-rated general health (n=834). * = statistically significant


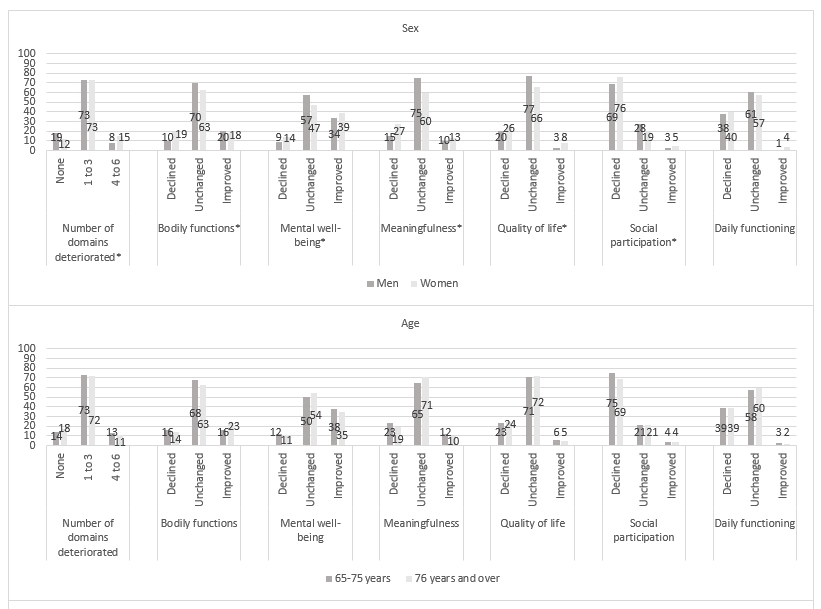


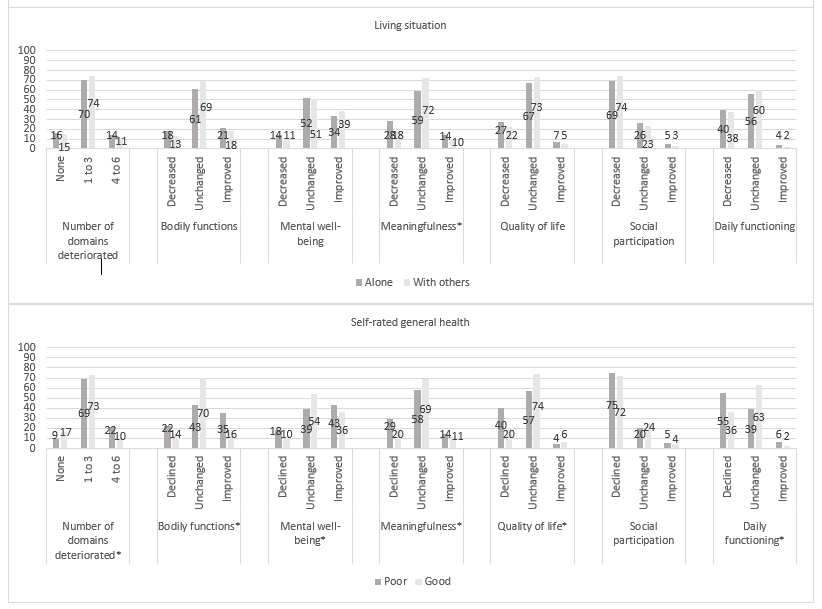

Supplement: Supplementary file 3 — Additional file 3. Self-rated change (%), compared to before the COVID-19 pandemic, in the six dimensions of Positive Health of older individuals living in the Netherlands depending on sex, age, living situation and self-rated general health (n=834). * = statistically significant. [file 12877_2021_2737_MOESM3_ESM.docx]
